# Connectiveness of Antimicrobial Resistance Genotype–Genotype and Genotype–Phenotype in the “Intersection” of Skin and Gut Microbes

**DOI:** 10.3390/biology14081000

**Published:** 2025-08-05

**Authors:** Ruizhao Jia, Wenya Su, Wenjia Wang, Lulu Shi, Xinrou Zheng, Youming Zhang, Hai Xu, Xueyun Geng, Ling Li, Mingyu Wang, Xiang Li

**Affiliations:** 1 Changsha Hospital for Maternal & Child Health Care Affiliated to Hunan Normal University, Changsha 410007, China; 202112535@mail.sdu.edu.cn; 2 State Key Laboratory of Microbial Technology, Microbial Technology Institute, Shandong University, Qingdao 266237, China; 202120381@mail.sdu.edu (W.S.); 202490900083@sdu.edu.cn (W.W.); 201912391@163.com (L.S.); zhengxinrou2000@163.com (X.Z.); zhangyouming@sdu.edu.cn (Y.Z.); haixu@sdu.edu.cn (H.X.); xygeng@sdu.edu.cn (X.G.); lingli@sdu.edu.cn (L.L.); 3 Shanghai Key Laboratory of Atmospheric Particle Pollution and Prevention (LAP), Shanghai 200443, China

**Keywords:** antibiotic resistance, antibiotic resistance phenotype, IncF plasmid, perianal skin, whole genome sequencing

## Abstract

The perianal skin is a unique “skin–gut” boundary that serves as a critical hotspot for the exchange and evolution of antibiotic resistance genes (ARGs). However, its role in the dissemination of antimicrobial resistance (AMR) has often been underestimated. To characterize the resistance patterns in the perianal skin environment of patients with perianal diseases and to investigate the drivers of AMR in this niche, a total of 51 bacterial isolates were selected from a historical strain bank containing isolates originally collected from patients with perianal diseases. All the isolates originated from the skin site and were subjected to antimicrobial susceptibility testing, whole-genome sequencing, and co-occurrence network analysis. The analysis revealed a highly structured resistance pattern, dominated by two distinct modules: one representing a classic Staphylococcal resistance platform centered around *mecA* and the *bla* operon, and a broad-spectrum multidrug resistance module in Gram-negative bacteria centered around *tet(A)* and predominantly carried by IncFIB and other IncF family plasmids. Further analysis pinpointed IncFIB-type plasmids as potent vehicles driving the efficient dissemination of the latter resistance module. Moreover, numerous unexplained resistance phenotypes were observed in a subset of isolates, indicating the potential presence of emerging and uncharacterized AMR threats. These findings establish the perianal skin as a complex reservoir of multidrug resistance genes and a hub for mobile genetic element exchange, highlighting the necessity of enhanced surveillance and targeted interventions in this clinically important ecological niche.

## Simple Summary

To explore the transmission risk of antibiotic resistance at the perianal skin in patients with perianal diseases, this study conducted a systematic and comprehensive analysis of 51 bacterial isolates derived from the perianal skin site. The results show that antibiotic resistance in the perianal skin environment is not randomly distributed. Instead, it exhibits a highly structured pattern dominated by two major antibiotic resistance modules. This environment has become a potential reservoir of transferable antibiotic resistance genes. The inconsistency between phenotype and genotype also suggests that this environment may harbor numerous unknown resistance genes or mechanisms. This study systematically revealed the phenotype–phenotype and phenotype–genotype connectiveness for antimicrobial resistance, providing a theoretical basis for the clinical treatment of patients with perianal diseases and the monitoring of antibiotic resistance transmission.

## 1. Introduction

The World Health Organization has identified antibiotic resistance as one of the major public health threats facing humanity in the twenty-first century [1]. Recent projections estimated that within the next 25 years, antibiotic resistance can lead to the deaths of approximately 39 million people. This threat is primarily driven by a broad spectrum of multidrug-resistant pathogens. The examples include Gram-negative bacteria such as carbapenem-resistant *Acinetobacter baumannii*, *Pseudomonas aeruginosa*, and extended-spectrum β-lactamase-producing Enterobacterales such as *Klebsiella pneumoniae* and *Escherichia coli*, as well as Gram-positive bacteria such as methicillin-resistant *Staphylococcus aureus* (MRSA) and vancomycin-resistant Enterococci (VRE) [2,3,4]. In response to the antibiotic resistance crisis, researchers have developed and implemented a wide range of strategies in an effort to control the spread and evolution of resistance [5,6,7,8]. The evolution of antibiotic resistance is remarkably rapid, not only driven by the selective pressure of antibiotic use, but also significantly facilitated by the horizontal transfer of ARGs via mobile genetic elements (MGEs) under various environmental conditions [9,10]. Plasmids exhibit high plasticity and can rapidly disseminate ARGs within bacterial populations both intra- and interspecies via horizontal gene transfer [11,12]. In multidrug-resistant plasmids, resistance genes often cluster to form resistance islands, which can adapt to environmental pressures by rapidly losing existing ARGs or acquiring new ones [13]. This dynamic genetic flexibility is a major contributor to the failure of clinical antibiotic therapies and presents a significant challenge for the surveillance and control of antibiotic resistance dissemination [14,15].

With the continuous advancement of sequencing technologies, a wide range of genomic and bioinformatic approaches have been employed to systematically analyze the reservoirs of ARGs in key ecological niches such as the gut, wastewater treatment plants, and livestock farms. These studies have comprehensively characterized the composition, mobility, and evolutionary mechanisms of ARGs. More importantly, they have revealed the associations between ARGs and MGEs [16,17,18,19,20]. However, despite the significant progress made in studying ARGs across various ecological niches, the application and exploration of the perianal skin—a critical microecological interface—remain largely unexplored.

The skin is the largest organ of the human body [21], and represents a complex microbial ecosystem jointly shaped by the host and its resident microbiota [22]. The microbial community of this ecosystem consists of bacteria and fungi, forming a stable yet dynamically regulated system [23]. Different regions of the skin are colonized by distinct microbial communities, and the composition of these communities is influenced by factors such as temperature, humidity, sebum levels, and pH [24]. The skin is in constant exchange with the external environment, and even in healthy individuals, it naturally harbors a diverse array of ARGs [25]. Dermatology is one of the hospital departments with the highest frequency of antibiotic use [26]. Such prolonged and low-dose antibiotic exposure creates an ideal selective environment for bacterial growth and the development of resistance [27]. Studies have reported that residents of nursing homes carry clonal populations of *Candida auris* and ESKAPE pathogens on their skin [28], suggesting that the skin may serve as a substantial and hidden reservoir of multidrug-resistant pathogens.

This risk is particularly pronounced in the perianal region, a unique anatomical niche. Perianal diseases are common conditions that occur around the anal region [29], encompassing a spectrum of symptoms with varying degrees of severity, including hemorrhoids [30], abscesses [31], and anal fistulas [32]. The perianal skin lies in close proximity to the anus, positioning it as a convergence zone between the skin and gut microbiota. The gut microbiota is widely recognized as the largest and most diverse reservoir of ARGs in the human body and is well known for carrying and exchanging a large number of ARGs via mobile genetic elements. This unique anatomical context, combined with the warm, moist, and semi-occluded environment of the perianal region, provides favorable conditions for bacterial growth [33,34]. The treatment of perianal diseases often involves the use of antibiotics to varying degrees. Under prolonged antibiotic selection pressure, the perianal region may become a high-risk hub for the accumulation and dissemination of multidrug-resistant bacteria [35]. However, the bacterial composition of the perianal skin and its role as a reservoir of antibiotic resistance remain poorly understood. To address this knowledge gap, the present study was conducted with the following specific objectives: (1) to characterize the composition and co-occurrence patterns of ARGs in the perianal skin microbiota; (2) to identify the key mobile genetic elements, particularly plasmids, responsible for ARG dissemination; and (3) to evaluate the concordance between resistance genotypes and clinically observed phenotypes.

## 2. Materials and Methods

### 2.1. Bacterial Isolates, Sequencing, and Data

The 51 bacterial isolates analyzed in this study were derived from a previously established strain repository in our laboratory. These strains were originally isolated from the perianal sites of patients with perianal diseases at Qilu Hospital (Qingdao). All the isolates used in this study were obtained specifically from skin sites. The 51 isolates used in this study were primarily composed of the following genera: *Staphylococcus* (*n* = 13), *Escherichia* (*n* = 16), *Klebsiella* (*n* = 9), *Proteus* (*n* = 1), *Enterococcus* (*n* = 10), and *Enterobacter* (*n* = 2). Some of the strains from this repository have been reported in previous studies. Detailed information on all the isolates, including species names, strain IDs, and references to related previous work, is provided in the Supplementary Materials (Table S1) [36,37]. The genomic DNA of the isolates was extracted using the Bacterial Genomic DNA Extraction Kit (Tiangen Biochemical Technology Co., Ltd., Beijing, China). Library preparation was performed using the barcode kit SQK-RBK114.96, and third-generation sequencing was conducted using the Nanopore P2solo with an R10.4.1 flow cell [38]. The sequencing data have been deposited in the NCBI GenBank database under accession numbers PRJNA1273337, PRJNA1125629, PRJNA1120246, PRJNA1125630, and PRJNA1119654.

### 2.2. Antimicrobial Susceptibility Testing

Antimicrobial susceptibility testing of all the isolates was performed using the Kirby-Bauer disk diffusion method. Overnight-grown single-colony bacterial isolates were inoculated into LB broth and incubated at 37 °C until reaching an optical density equivalent to 0.5 McFarland. The bacterial suspension was then inoculated onto Mueller–Hinton (MH) agar plates, and antibiotic disks were placed on the agar surface within 15 min. The plates were incubated at 37 °C for 16–18 h. Antimicrobial susceptibility testing was performed using 22 antibiotics for 27 *Enterobacteriaceae* isolates and 1 *Morganellaceae* isolate, with *Escherichia coli* ATCC 26922 used as the quality control strain. For 10 *Enterococcaceae* isolates, 12 antibiotics were tested, and for 13 *Staphylococcaceae* isolates, 6 antibiotics were tested; *Staphylococcus aureus* ATCC 25923 was used as the quality control strain for both groups. Detailed information on the antibiotics used is provided in Table S4. Inhibition zone diameters were measured and interpreted according to the CLSI M100 guidelines [39]. Polymyxin susceptibility testing was conducted using the broth microdilution method, as recommended by the CLSI M100 standard.

### 2.3. Bioinformatic Analysis

ARGs were identified from all the genome assemblies using AMRfinder version 3.11.26 [40]. Hits were considered positive if the percentage identity was ≥90% and the template coverage was ≥60%. Identified ARGs were categorized based on the class of antibiotics. Plasmid replicon types were identified from genome assemblies using Plasmidfinder version 2.1.1 with an identity threshold of ≥95% and a coverage threshold of ≥80% [41]. The genome annotation was carried out using either the Prokaryotic Genome Annotation Pipeline or Prokka (v1.14.6) [42]. The genomic location (chromosome or plasmid) of ARG was inferred as follows: an ARG was considered plasmid-borne if its corresponding contig also harbored one or more known plasmid replicons. An ARG was considered chromosome-borne if it was lacking any detectable plasmid replicons. The overall location category for each gene type (‘Plasmid Only’, ‘Chromosome Only’, or ‘Both’) was determined by summarizing its inferred locations across all the isolates carrying that gene. Bar charts for plasmid carriage rates, plasmid replicon diversity, and nested donut charts were generated using matplotlib 3.10.0 and pandas libraries 2.2.3 in Python 3.12.0. All the parameters were kept at their default settings.

### 2.4. Phenotype–Genotype Concordance Analysis

Phenotype–genotype concordance data were visualized as heatmaps using the Python programming language combined with the pandas and seaborn libraries. Based on five predefined phenotype–genotype concordance categories, corresponding to values from 1 to 5, a custom color mapping (red, blue, orange, green and white) was set to represent different categories. The heatmap was generated using the heatmap function of the seaborn library. Concordance categories were defined as (1) Phenotype and Genotype positive; (2) Phenotype and Genotype negative; (3) Phenotype positive, Genotype negative; (4) Phenotype negative, Genotype positive; and (5) Others or Not tested. Heatmaps were generated using the seaborn library 3.13.2 in Python.

To quantitatively assess the concordance between the phenotypic antibiotic susceptibility results and the presence of corresponding resistance genes, Cohen’s Kappa statistic was calculated for each antibiotic. Only drug–strain data pairs with both phenotypic and genotypic information were included in the concordance analysis. The Kappa values were interpreted based on the classic criteria proposed by Landis and Koch [43]. All the statistical analyses were performed using the scikit-learn library in Python 3.12.0.

### 2.5. ARG Co-Occurrence Network Analysis

The co-occurrence network was constructed as an undirected weighted graph using the NetworkX library 3.4.2. In this network, each node represents a unique ARG, and an edge is drawn between two nodes if the corresponding genes co-occur in at least one sample. The weight of each edge is defined as the total number of samples in which the two genes co-occur. To optimize the network structure and highlight more robust associations, a co-occurrence frequency threshold (threshold = 3) was applied, retaining only edges with weights equal to or greater than this value for network construction. After the initial network was built, all the isolated nodes (i.e., nodes with no connections to other nodes) were removed to focus the analysis on core co-occurrence modules. Co-occurrence “clusters” of genes were defined by identifying connected components within the network. Network visualization was performed using Cytoscape version 3.10.3 [44].

### 2.6. Correlation Analysis Between Antibiotic Categories and Plasmid Replicon Types

Associations between ARG and specific plasmid replicon types were assessed by counting the number of unique isolates in which they co-localized on the same contig. The results were visualized using heatmaps (with Seaborn) and bipartite network graphs. The creation of bipartite network graphs was performed using Cytoscape version 3.10.3.

### 2.7. Use of GenAI

Gemini 2.5pro and ChatGPT 3.5 models were used for language translation and polishing, and for generating code for preparing illustrations. All the texts and codes that were generated/modified were manually verified. Detailed code is provided in the Supplementary Materials.

## 3. Results

### 3.1. Strains and Overview of ARGs

The 51 bacterial isolates analyzed in this study were derived from a previously established strain repository in the laboratory. These isolates, all derived from skin sites, representing various common species found in the perianal microbiota, were classified into six genera, ranked by abundance as follows: *Escherichia*, *Staphylococcus*, *Enterococcus*, *Klebsiella*, *Enterobacter*, and *Proteus*. Whole-genome analysis revealed substantial variation in the numbers of ARGs carried by different strains, even among isolates of the same genus or species. In addition, the genomic localization of these ARGs varied markedly across strains.

**Figure 1:**
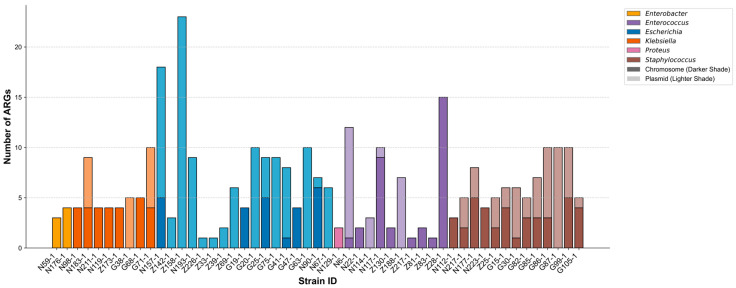
Distribution of antimicrobial resistance gene (ARG) across individual bacterial strains. Each bar represents a unique bacterial isolate, with the total height of the bar representing the total number of ARG detected in the strain. The bars are segmented to show the number of ARG predicted to be located on the chromosome (dark colored bottom segment) and on plasmids (light colored top segment). The color of each bar denotes the bacterial genus and the corresponding genomic location of ARG. Data are shown for *n* = 51 strains.

ARGs were predicted from whole-genome sequences with AMRFinderPlus. Plasmid typing was performed with PlasmidFinder. Figure 1 summarizes the total number of ARGs detected in each strain and their distribution between chromosomal and plasmid locations. Substantial variability was observed in the number of ARGs carried by different strains. For instance, within the same species, *Escherichia coli* Z158-1 carried as many as 23 ARGs, whereas *E. coli* Z226-1 carried only one. This intra-species variability was particularly pronounced in *Escherichia* and *Enterococcus*. Moreover, the analysis revealed that in *Escherichia*, a notably high proportion of ARGs were plasmid-borne.

**Figure 2:**
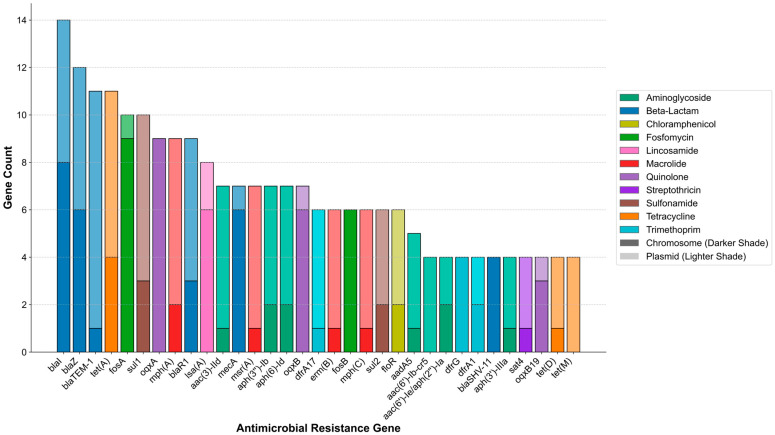
Distribution and genomic location of the 33 most frequent ARGs. Each bar represents a specific ARG, presented in descending order of the total number of unique isolates in which it was detected. The total height of each bar indicates the number of isolates carrying the gene. The bars are segmented to show the number of isolates where the gene was identified on chromosomes vs. on plasmids. The color of each bar corresponds to the antibiotic class to which the ARG confers resistance. Data are compiled from *n* = 90 genes.

A further analysis was conducted on the types of non-redundant ARGs carried by the bacterial strains. Figure 2 displays the genomic localization of the top 33 most frequently occurring ARGs, with the top nine ranked by prevalence. Among them, β-lactam resistance genes were the most abundant, followed by those conferring resistance to tetracyclines and fosfomycin. The three most frequently detected ARGs were *blaI*, *blaZ*, and *bla*_TEM-1_, all of which are associated with β-lactam resistance. The *blaI* and *blaZ* genes are typically found in Gram-positive bacteria and function as components of the β-lactamase regulatory system, whereas *bla*_TEM-1_ is predominantly detected in Gram-negative bacteria and represents one of the most common plasmid-mediated resistance genes, known for its high potential for horizontal gene transfer. All three genes were highly prevalent in our dataset and were located on both chromosomes and plasmids, suggesting a potential risk of cross-genus dissemination of resistance determinants within the perianal skin microbiota. The analysis also found that the distribution of these ARGs between chromosomes and plasmids is uneven. For example, *bla*_TEM-1_ and *tet(A)* are more commonly found on plasmids, which is consistent with their known role in horizontal gene transfer. In contrast, *blaI* and *blaZ* show a more balanced distribution or a slight bias towards chromosomal localization. This suggests that different β-lactam ARGs possess different mobilization potentials. In addition, genes such as *fosA* and *oqxAB* were predicted to be more frequently located on the chromosome, which may be attributed to their intrinsic presence in the chromosomes of certain bacterial species. However, they are also capable of mobilizing from the chromosome to plasmids via mobile genetic elements, thereby contributing to the dissemination of resistance.

### 3.2. ARG Co-Occurrence Network Reveals a Highly Modular Resistance Structure

**Figure 3:**
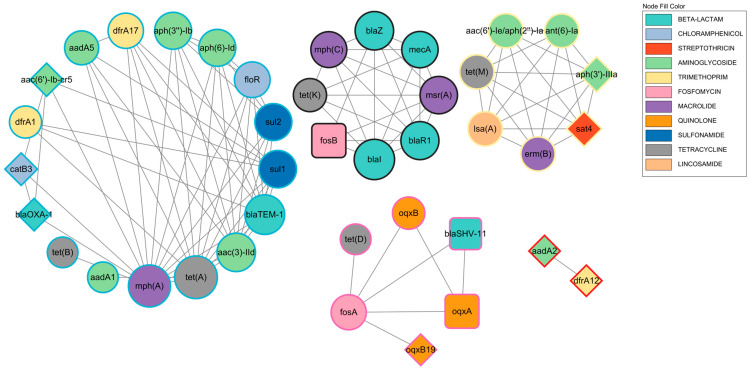
Co-occurrence network of ARGs. This network illustrates the associations between ARGs with a co-occurrence frequency of 3 or more across all the isolates. In the graph, each node represents a distinct ARG, and an edge connecting two nodes indicates a co-occurrence relationship between the corresponding genes. The fill color of each node indicates the antibiotic class to which the ARG belongs. Node shape represents the overall genomic location in the dataset (circle: present in both plasmid and chromosome; triangle: plasmid only; square: chromosome only). Node size is proportional to its weighted degree, with larger nodes indicating stronger co-occurrence associations in the network. The border color of nodes distinguishes different co-occurrence clusters, with nodes sharing the same border color belonging to the same functional module. The black outer circle represents Cluster 1, the blue outer circle represents Cluster 2, the yellow outer circle represents Cluster 3, the pink outer circle represents Cluster 4, and the red outer circle represents Cluster 5.

In this study, a total of 90 unique ARGs were identified. To uncover patterns of co-occurrence among these genes, a co-occurrence network was constructed based on their presence across samples. In this network, each node represents a distinct ARG, and an edge is drawn between two nodes if the corresponding genes co-occurred in the same isolate at least three times (co-occurrence threshold ≥ 3). The final network consisted of 40 nodes and 107 edges, which were organized into five distinct connected components. This modular structure indicates that the co-occurrence of ARG is not random but instead exhibits well-defined organizational patterns (Figure 3).

The association between gene co-occurrence and genomic locations was further explored by quantifying the connections between nodes of different location types within the network. The corresponding results are summarized in Table S5. The results showed that the network connections were predominantly driven by mobile genes that were found on both plasmids and chromosomes, with internal connections among these genes accounting for 80.4% (86/107) of the total edges. Most notably, there were no direct connections between the genes exclusively located on chromosomes and those exclusively located on plasmids (0/107), suggesting that there may be certain barriers to gene exchange or co-transfer between these two groups. However, the genes that are present on both plasmids and chromosomes were connected to both of these gene groups, suggesting that they serve as critical bridges within the network.

To identify key genes within the ARG co-occurrence network, a centrality analysis of the nodes was performed (Table S2). The results showed that *tet(A)* was the most central node in the network, with the highest weighted degree (weighted degree = 60) among all the genes. This indicates that *tet(A)* functions as a critical hub in the ARG co-occurrence network within this sample set. Its high centrality is driven by both extensive connectivity (degree = 12) and strong average interaction strength (average edge weight = 5.0). Other important hub genes included *mph(A)* and *blaI*. The *mph(A)* gene exhibited the highest degree in the network (degree = 14), suggesting that it is the most socially connected gene, interacting with the largest number of different gene types. In contrast, *blaI* was characterized by an exceptionally high average edge weight (average edge weight = 7.0), indicating that it forms particularly stable and high-frequency co-occurrence relationships with a small number of genes—serving as a “selective and stable” core node.

Notably, the topology of the network exhibited pronounced centralization and modularity. A detailed centrality analysis revealed that all of the top 10 hub genes (ranked by weighted degree) belonged exclusively to the two largest connected components—Cluster 1 and Cluster 2—indicating that the ARG network in this microbial environment is dominated by these two core modules. Cluster 2 is the largest and most functionally diverse module within the network, representing a typical broad-spectrum multidrug resistance platform. The primary hub gene of the network, *tet(A)*, serves as the backbone of this cluster. Cluster 2 also includes several other critical hub genes, such as *mph(A)*, *bla*_TEM-1_, *aac(3)-IIc*, *sul1*, and *sul2*. The combination of these genes equips this module with resistance to multiple clinically important antibiotic classes, including tetracyclines, macrolides, β-lactams, aminoglycosides, and sulfonamides. In sharp contrast to the broad-spectrum profile of Cluster 2, Cluster 1 represents a more functionally specialized and cohesive resistance module. The core members of this cluster include *blaI*, *blaZ*, and *blaR1*, which together constitute the classical *bla* operon responsible for regulating β-lactamase expression in *Staphylococcus* species. Notably, *blaI* exhibited the highest average edge weight among all top-ranked genes, indicating an exceptionally tight functional synergy or co-regulatory relationship with other genes within the cluster. Most importantly, this cluster also contains the *mecA* gene, a marker gene of methicillin-resistant *Staphylococcus aureus* (MRSA), which confers resistance to virtually all β-lactam antibiotics. The *mecA* gene is carried by a large mobile genetic element known as the SCC*mec* cassette, which often integrates additional resistance determinants, further enhancing the multidrug resistance potential of this module.

### 3.3. Plasmid Characteristics and Their Associations with ARGs

**Figure 4:**
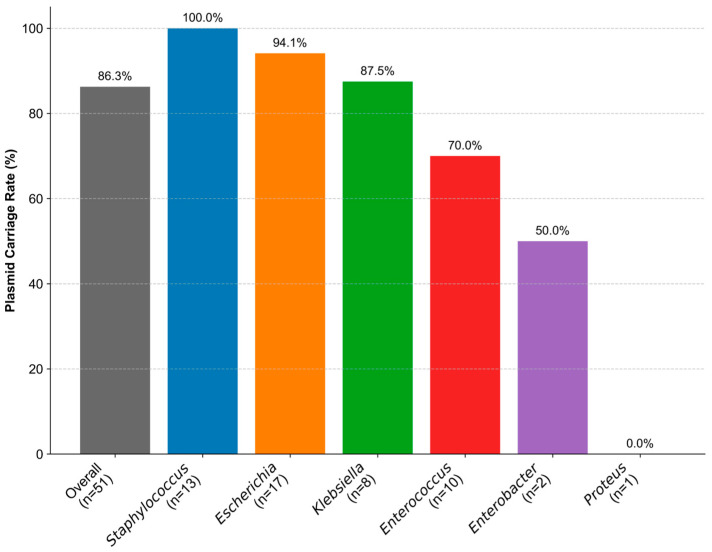
Plasmid carriage rates in bacterial isolates. This figure shows the percentage of isolates harboring detectable plasmids, shown for the overall population and for each bacterial genus identified. The genera are presented in descending order of their plasmid carriage rate. The number of isolates (*n*) analyzed for each category is indicated below the category name on the x-axis.

Based on the observations from the co-occurrence network analysis—where plasmid-encoded ARGs play a critical role in linking different bacterial genera and resistance gene clusters—the characteristics of plasmids and their associations with ARGs were further investigated to gain deeper insights into their potential mechanisms in mediating resistance dissemination. As a first step, the overall plasmid carriage rates across all the strains and their distribution among different genera were analyzed (Figure 4). Plasmids were detected in the majority of strains, with an overall carriage rate of 86.3%. Notably, plasmids were almost universally present in certain genera, such as *Staphylococcus* and *Escherichia*, with *Staphylococcus* exhibiting a 100% carriage rate. In contrast, plasmid detection was lower in *Enterobacter* (50%, 1/2), and no plasmids were identified in *Proteus* (0/1).

**Figure 5:**
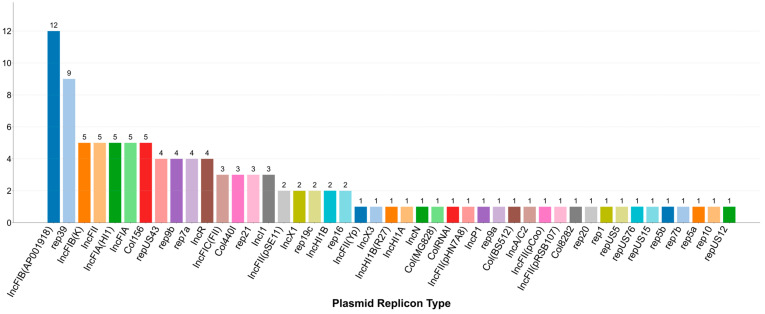
Diversity and frequency of detected plasmid replicon type across bacterial isolates. Each bar represents a unique plasmid replicon type identified in the study cohort, ordered from left to right by decreasing frequency of detection. The height of each bar corresponds to the number of unique bacterial isolates in which that specific replicon type was detected.

The plasmids detected in the samples were then subjected to replicon typing and frequency analysis. (Figure 5). The findings indicate that plasmid dissemination is not evenly distributed, but instead dominated by a small number of prevalent replicon types. Among the 39 distinct replicon types identified, IncFIB (AP001918) was the most dominant, detected in 12 strains. This was followed by the IncR replicon, which was found in 9 strains. In addition, multiple members of the IncFII family and several Col-type plasmids were also relatively common, each found in 4 to 5 strains. These results suggest that plasmids belonging to the IncF families represent key plasmid groups within this microbial community.

**Figure 6:**
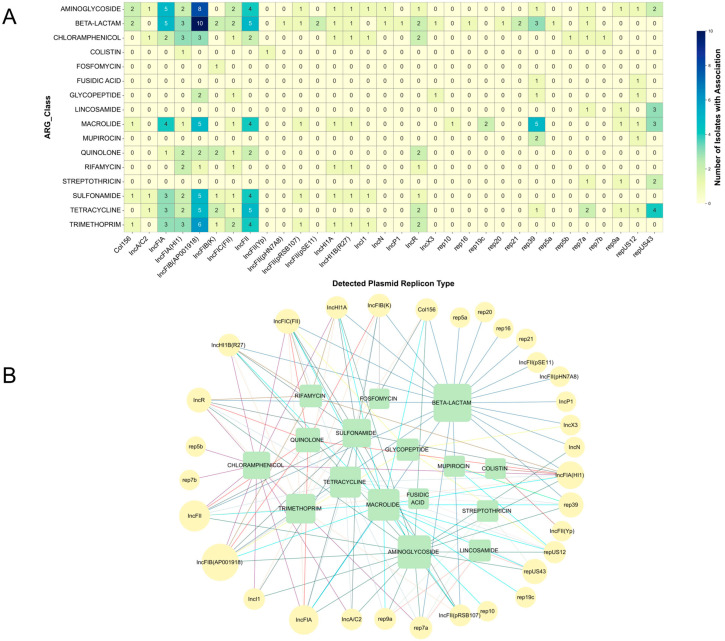
Association patterns between plasmid replicon types and classes of ARG. (**A**) Heatmap illustrating the co-occurrence frequency of specific plasmid replicon types with different ARG classes. Cell color intensity and the annotated number represent the count of unique isolates in which a given plasmid replicon type was found on the same contig as an ARG belonging to the indicated antibiotic class. (**B**) Bipartite network representation of the associations between plasmid replicon types and ARG classes. Nodes are distinguished by type: plasmid replicon types are depicted as yellow circles and ARG classes as green squares. Node size is proportional to its weighted degree. The color of the edges corresponds to the category of the antibiotic resistance genes.

To further investigate the associations between different ARG functional categories and specific plasmid replicon types, the co-occurrence frequencies between ARG categories and plasmid replicon types were analyzed (Figure 6). The heatmap analysis (Figure 6A) revealed strong association patterns between certain ARG categories and specific plasmid replicon types. Notably, aminoglycoside and β-lactam resistance genes exhibited the highest co-occurrence frequency with IncFIB (AP001918). Additionally, IncFIB (AP001918) showed significant co-occurrence with macrolide and tetracycline resistance genes, highlighting its potential role as a multidrug resistance carrier. This complex association is more intuitively illustrated in the network diagram (Figure 6B). The analysis clearly shows that β-lactam resistance genes represent the node with the highest total association strength among all the ARG functional categories, connecting to as many as 22 different plasmid replicon types. This indicates that β-lactam resistance genes not only exhibit strong association strength but also are associated with a broad and diverse range of transmission vectors. Following closely is the aminoglycoside category, which connects to 18 different plasmid types. Other ARG functional categories demonstrating significant association strength include tetracycline, trimethoprim, and sulfonamide resistance genes. Among the plasmid replicon types, IncFIB (AP001918) holds a core position with the highest weighted degree of 48 (Table S3) and connects to 11 different ARG functional categories. This further confirms its key role as a “super-spreader” of multidrug resistance (MDR) genes. Other members of the IncF family, such as IncFII and IncFIA, also exhibit strong associative capabilities, underscoring the dominant role of IncF family plasmids in mediating the coordinated dissemination of multiple resistance phenotypes.

### 3.4. Discrepancies Between Phenotypic and Genotypic Resistance Reveal the Potential of Perianal Skin as a Reservoir for Unrecognized Resistance Mechanisms

**Figure 7:**
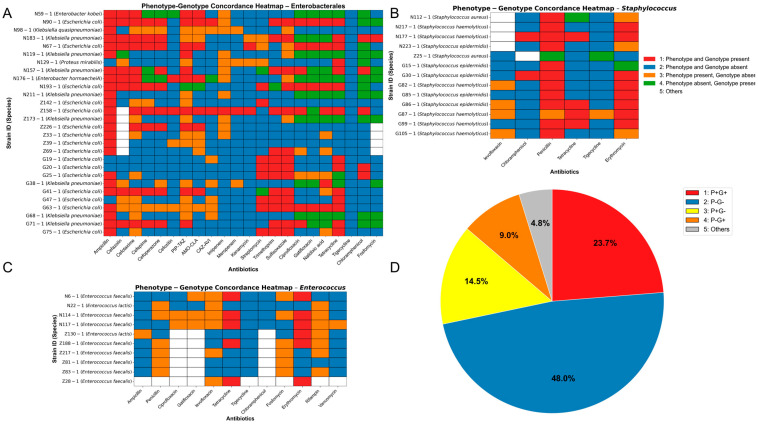
Phenotype–genotype concordance analysis of antimicrobial resistance across major bacterial groups. (**A**–**C**) Heatmaps displaying the phenotype–genotype concordance for isolates belonging to (**A**) Enterobacterales, (**B**) *Staphylococcus*, and (**C**) *Enterococcus*. Each row represents a unique strain, and each column represents an antibiotic. Cells are colored according to the concordance category. (**D**) The pie chart summarizes the overall distribution of phenotype–genotype concordance categories from the three bacterial groups.

Plasmid analysis revealed that multiple resistance-associated replicon types were widely distributed among perianal skin isolates, suggesting this site may serve as an active reservoir and dissemination platform for resistance determinants. However, the presence of ARGs does not necessarily indicate functional expression or phenotypic resistance, and vice versa. To further assess phenotypic features of isolated strains, disk diffusion susceptibility tests were performed on three major bacterial groups (Enterobacterales, *Enterococcus*, and *Staphylococcus*), and the results were compared with whole-genome sequencing data to evaluate genotype–phenotype concordance (Table S4) (Figure 7A–D).

For Enterobacterales isolates (Figure 7A), a generally high level of genotype–phenotype concordance was observed, particularly for β-lactam antibiotics such as ceftriaxone, ampicillin, and aztreonam. However, increased discordance was noted for tetracyclines and sulfonamides. For *Enterococcus* (Figure 7C), a higher proportion of inconsistent patterns emerged, especially for fluoroquinolones and aminoglycosides. *Staphylococcus* isolates (Figure 7B) showed marked inter-strain heterogeneity. Notably, some *mecA*-positive strains remained susceptible in disk diffusion tests. Although approximately 72% of the results fell into fully concordant categories, around 24% of the isolates exhibited varying degrees of discordance (Figure 7D), suggesting the presence of unannotated and non-classical resistance mechanisms.

To provide a more rigorous statistical evaluation of the concordance between phenotype and genotype, while correcting for agreement by chance, Cohen’s Kappa statistic was calculated for each antibiotic across the three bacterial groups (Table S5). The results of the Kappa analysis revealed that the discordance was more complex and pronounced than suggested by the simple concordance statistics. Although some antibiotics showed relatively high raw concordance rates, their Kappa values were only moderate, fair, or even poor. For example, low concordance (K < 0.4) was observed in 40.9% of antibiotics tested in Enterobacterales, 66.7% in *Staphylococcus*, and up to 80% in *Enterococcus*. These quantitative results statistically confirm the complex relationship between the presence of resistance genes and their actual phenotypic expression, indicating that the mere presence of a gene does not reliably predict its functional expression.

## 4. Discussion

This study focused on bacteria present in the perianal skin and systematically revealed the resistance patterns within this anatomical site. First, ARGs present at this site are not randomly distributed, but instead exhibit a highly modular organization. Notably, the resistance landscape in this environment is dominated by two functionally distinct modules: one is a staphylococcal resistance module centered around *mecA*, originating from the skin microbiota; the other is a broad-spectrum multidrug resistance module centered around *tet(A)*, originating from the gut microbiota. Secondly, the study found that plasmids of the IncFIB family are key vectors mediating the dissemination of the latter. Finally, the study revealed that a large number of strains in this environment exhibit discordance between genotype and phenotype, highlighting the complexity of the genotype–phenotype relationship in the perianal skin and suggesting the potential presence of numerous uncharacterized resistance mechanisms. These findings establish the perianal skin as a complex, dynamic, and clinically significant hub for ARG exchange.

The perianal skin represents a unique and microbiologically complex region of the human body [45]. Due to its distinct anatomical and physiological location, the perianal area is well known as a microbial hotspot. The analysis revealed that the resistome within this unique ecological niche is not composed of scattered genes arranged randomly, but rather exhibits a highly structured organization, dominated by two distinct and clinically significant resistance modules. This finding suggests that the core of the network is governed by a small set of hub genes with diverse functions and distinct connectivity patterns. The ARG co-occurrence network analysis identified two major ARG clusters. Cluster 1 is centered around the *mecA* gene and the *blaI*-*blaZ*-*blaR1* operon, accompanied by genes such as *msr(A)* and *mph(C)*, which clearly indicate resistance traits characteristic of staphylococci, particularly methicillin-resistant *Staphylococcus aureus* (MRSA). This cluster likely originates from the evolution of resident skin flora under specific pathological conditions. Its composition is highly consistent with the resistome of coagulase-negative staphylococci, such as *Staphylococcus epidermidis*, which are commonly isolated from other skin sites, including the axilla, nasal cavity, and groin. These sites are well known as reservoirs of SCC*mec* elements [46,47,48]. Although the *mecA* identified in this study were located in *Staphylococcus epidermidis* and *Staphylococcus haemolyticus*, there is a strong possibility that they could be transferred to the *S. aureus* species within the same genus [49]. In stark contrast to this, Cluster 2 is dominated by *tet(A)* and features a broad collection of resistance genes such as *mph(A)*, *bla*_TEM-1_, *sul1*, as well as various aminoglycoside and trimethoprim resistance genes. It constitutes a typical broad-spectrum multidrug resistance module closely associated with Gram-negative bacteria, and serves as a hallmark feature of the resistome of *Enterobacteriaceae* colonizing the gut [50,51,52]. This multi-module resistance pattern reflects the complexity of the perianal skin as a microbial convergence zone between the skin and the gut. During the treatment of perianal diseases, this environment is frequently exposed to antibiotic pressure, which facilitates the rapid acquisition and dissemination of these highly integrated genetic elements between both microbial communities.

Further analysis confirmed that plasmids are the primary carriers of these ARG modules. Notably, among the diverse plasmid replicon types detected, IncFIB (AP001918) and other members of the IncF family dominated. IncF family plasmids are well-recognized epidemic plasmids that have facilitated the dissemination of key resistance determinants, such as ESBLs, in both clinical and community settings [53,54]. While previous studies have mostly focused on the role of these plasmids in the gut or hospital settings, our findings highlight the perianal skin as a key site for their persistence and mobilization. The network analysis linking ARG functional categories to plasmid vectors quantitatively demonstrated that β-lactam and aminoglycoside resistance is primarily disseminated via these IncF-family plasmids in a highly efficient and coordinated manner. These findings indicate that IncF plasmids hold a clear advantage both in terms of abundance and functional capacity. They harbor resistance genes against multiple clinically important antibiotics and form complex co-occurrence networks that connect distinct bacterial genera and resistance gene clusters. As such, they serve as central hubs for the dynamic exchange and mobilization of multidrug resistance elements. This provides direct evidence for understanding the formation and efficient dissemination of Cluster 2 in this analysis.

In addition, a substantial proportion of isolates exhibited discordance between genotypic and phenotypic resistance profiles. The presence of phenotypic resistance in the absence of known resistance genes may suggest the existence of novel resistance genes not yet included in the current databases, previously uncharacterized resistance mechanisms, or non-genetic regulatory pathways. Conversely, the presence of resistance genes without corresponding phenotypic resistance may be due to these genes being silent under the given environmental conditions; once activated, they could exert resistance functions and alter the phenotypic profile. This phenomenon is not unique to our study and highlights the complexity of antibiotic resistance regulation [55,56]. Given the abundance of mobile genetic elements in perianal skin, these unknown or “silent” ARGs, once mobilized and activated, may become significant contributors to future antibiotic resistance threats.

The findings of this study have potential clinical and public health significance. Firstly, this study reveals that the perianal skin serves as an overlooked reservoir of multidrug-resistant bacteria and mobile ARGs, suggesting that targeted screening and decolonization interventions at this site may offer an effective strategy for preventing serious complications such as surgical site infections. Secondly, the modular resistance patterns identified in the perianal skin provide important theoretical support for the empirical treatment of perianal infections. These findings can help clinicians avoid known resistance pathways and develop more targeted initial combination therapies, thereby improving the success rate of clinical treatment. Moreover, this study identified a substantial number of unexplained resistance phenotypes, suggesting that the current diagnostics based on known resistance genes may fail to detect novel or cryptic resistance mechanisms hidden in this niche. This finding underscores the importance of continued resistance gene mining and predictive research, and may drive the development of next-generation rapid diagnostic tools to address these emerging AMR threats.

Despite providing comprehensive insights into the diversity and dissemination characteristics of ARGs and plasmids in the perianal skin microbiota of patients with perianal diseases, this study has certain limitations. The analysis in this study focused on cultivable bacterial isolates, which may exclude bacteria that cannot grow under standard laboratory conditions. Therefore, the observed resistance network may not fully represent the diversity of ARGs within the perianal skin microbiome. Moreover, the strain database analyzed in this study was derived from a single medical institution. Additionally, the overall sample size remains limited, and some bacterial genera are underrepresented, potentially leading to an underestimation of the diversity of rare resistance mechanisms. Future studies should include larger cohorts, multi-center sampling, and broader clinical contexts to validate and extend the resistance dissemination patterns revealed in this study. In addition, metagenomic analyses should be conducted to overcome the limitations associated with the culture-dependent approach used in this work. Furthermore, given the substantial genotype–phenotype discrepancies observed, future efforts should focus on uncovering and characterizing novel and previously unrecognized resistance mechanisms.

## 5. Conclusions

In conclusion, this study reveals that ARGs in the perianal skin are highly structured and organized into two dominant modules: one centered around *mecA*, representing a staphylococcal resistance module, and the other centered around *tet(A)*, representing a broad-spectrum Gram-negative resistance module. Furthermore, the analysis indicates that plasmids of the IncFIB family are the primary vectors associated with the dissemination of this multidrug resistance module. In addition, the significant discordance between genotypes and phenotypes suggests the presence of resistance mechanisms in this niche that remain unknown. Overall, these findings establish the perianal skin as a complex and clinically significant hub for the accumulation and exchange of mobile resistance elements, underscoring its importance in AMR surveillance and infection control.

## Data Availability

The original data presented in the study are openly available in Genbank under accession numbers PRJNA1273337, PRJNA1125629, PRJNA1120246, PRJNA1125630, and PRJNA1119654.

## Supplementary Materials

The following supporting information can be downloaded at https://www.mdpi.com/article/10.3390/biology14081000/s1, Table S1: Bacterial strain information table; Table S2: Weighted co-occurrence table of antibiotic resistance genes in the network; Table S3: Weights of association between antibiotic resistance genes and plasmid replicons; Table S4: Antimicrobial susceptibility tests. Table S5: Quantification of edge types by genomic context of connected ARGs in the co-occurrence network. Table S6: Kappa statistics. Supplementary Document Code.

## Abbreviations

The following abbreviations are used in this manuscript:

ARGs	antibiotic resistance genes
AMR	antimicrobial resistance
MGEs	mobile genetic elements
ESBLs	extended-spectrum beta-lactamases
