# Supplementary material for: Connectiveness of Antimicrobial Resistance Genotype–Genotype and Genotype–Phenotype in the “Intersection” of Skin and Gut Microbes
Source: Biology (Basel). 2025 Aug 5;14(8):1000. doi: 10.3390/biology14081000 (PMC12383831; doi:10.3390/biology14081000)
Supplement: Supplementary file 1 [file biology-14-01000-s001.zip › supplementary code.pdf]

AMR plasmid and chromosome counts statistics

```
import pandas as pd
import matplotlib.pyplot as plt
import matplotlib.colors as mcolors
from matplotlib.patches import Patch

# Try to read the Excel file
try:
    df = pd.read_excel('AMRnumber.xlsx')
    print("Successfully read the Excel file.")
except FileNotFoundError:
    print("Error: 'AMRnumber.xlsx' file not found. Please check the filename and path.")
    exit()

# Clean and preprocess data
df['Genus'] = df['Genus'].str.strip().str.capitalize()
df['Chromosome_AMR'] = df['Chromosome_AMR'].fillna(0)
df['Plasmid_AMR'] = df['Plasmid_AMR'].fillna(0)

# Define genus color mapping
genus_color_map = {
    'Enterobacter': '#e69f00',
    'Klebsiella': '#d55e00',
    'Escherichia': '#0072b2',
    'Proteus': '#cc79a7',
    'Enterococcus': '#8167a9',
    'Staphylococcus': '#8c564b',
    'Other_Genus': '#cccccc'
}

# Assign color shades based on genus
def get_genus_colors(row):
    base_color = genus_color_map.get(row['Genus'], genus_color_map['Other_Genus'])
    return mcolors.to_rgba(base_color, 1.0), mcolors.to_rgba(base_color, 0.6)

df[['Color_dark', 'Color_light']] = df.apply(get_genus_colors, axis=1, result_type='expand')

# Set global plot parameters
plt.rcParams.update({
    'font.size': 12, 'font.family': 'sans-serif',
    'font.sans-serif': ['Arial', 'Helvetica'],
    'axes.labelweight': 'bold', 'axes.titleweight': 'bold'
})
```

```
# Create the figure and axes
fig, ax = plt.subplots(figsize=(20, 7))
ax.grid(axis='y', linestyle='--', alpha=0.7, zorder=0)
ax.spines['top'].set_visible(False)
ax.spines['right'].set_visible(False)

# Plot stacked bars for chromosome and plasmid ARGs
ax.bar(df['SampleID'], df['Chromosome_AMR'], color=df['Color_dark'], edgecolor='black',
label='On Chromosome')
ax.bar(df['SampleID'], df['Plasmid_AMR'], bottom=df['Chromosome_AMR'],
color=df['Color_light'], edgecolor='black', label='On Plasmid')

# Create legend patches for genera and AMR locations
legend_patches = []
plotted_genera = df['Genus'].unique()
for genus in sorted(plotted_genera):
    if genus in genus_color_map:
        patch_color = genus_color_map.get(genus)
        genus_italic = rf"$\it{{{genus}}}$"
        legend_patches.append(Patch(facecolor=patch_color, label=genus_italic,
edgecolor='dimgray'))

legend_patches.append(Patch(facecolor='dimgrey', alpha=1.0, label='Chromosome (Darker
Shade)'))
legend_patches.append(Patch(facecolor='darkgrey', alpha=0.6, label='Plasmid (Lighter Shade)'))

# Add labels and title
ax.set_xlabel('Strain ID', fontsize=14)
ax.set_ylabel('Number of ARGs', fontsize=14)
ax.set_title('ARG Distribution per Strain (Chromosome vs Plasmid)', fontsize=16)

# Adjust tick labels
plt.xticks(rotation=45, ha='right', fontsize=11)
ax.tick_params(axis='y', labelsize=11)

# Add the legend
ax.legend(
    handles=legend_patches,
    title='Legend',
    bbox_to_anchor=(1.02, 1),
    loc='upper left',
    fontsize=11,
    title_fontsize=13
)
```

```
plt.tight_layout(rect=[0, 0, 0.88, 1])

# Save the figure
output_filename_tif = "ARG_Distribution_per_Strain_Final.tif"
try:
    plt.savefig(output_filename_tif, dpi=300, format='tiff', bbox_inches='tight')
    print(f"Figure saved as high-resolution TIF file: {output_filename_tif}")
except Exception as e:
    print(f"Error occurred while saving TIF file: {e}")

plt.show()
```

Phenotype-Genotype Heatmap Analysis

```
import pandas as pd
import seaborn as sns
import matplotlib.pyplot as plt
from matplotlib.colors import ListedColormap, BoundaryNorm
import matplotlib.patches as mpatches

def main():
    print("--- Starting heatmap data processing ---")
    try:
        df = pd.read_excel('heatmap_Enterococcus.xlsx', usecols="A:N")
        print("Successfully read the Excel file.")
        df['Strain_Species'] = df['Strain'] + ' (' + df['Species'] + ')'
        df.set_index('Strain_Species', inplace=True)
    except FileNotFoundError:
        print("Error: File 'heatmap_Enterococcus.xlsx' not found.")
        return
    except Exception as e:
        print(f"An error occurred while reading or processing data: {e}")
        return

    antibiotics = df.columns[2:]
    heatmap_data = df[antibiotics].apply(pd.to_numeric, errors='coerce').fillna(0).astype(int)

    colors = ['#d62728', '#1f77b4', '#ff7f0e', '#2ca02c', '#ffffff']
    cmap = ListedColormap(colors)
    bounds = [0.5, 1.5, 2.5, 3.5, 4.5, 5.5]
    norm = BoundaryNorm(bounds, cmap.N)

    plt.rcParams.update({
        'font.family': 'sans-serif',
```

```
        'font.sans-serif': ['Arial', 'Helvetica'],
        'axes.labelweight': 'bold',
        'axes.titleweight': 'bold',
        'axes.unicode_minus': False
    })

    print("Drawing heatmap...")
    cell_width = 0.6
    cell_height = 0.5
    fig_width = cell_width * len(antibiotics) + 4
    fig_height = cell_height * len(heatmap_data)
    fig, ax = plt.subplots(figsize=(fig_width, fig_height))

    sns.heatmap(
        heatmap_data,
        cmap=cmap,
        norm=norm,
        cbar=False,
        linewidths=0.5,
        linecolor='black',
        ax=ax
    )

    title_string = r'$\mathbf{Phenotype-Genotype\ Concordance\ Heatmap\ –\ }\mathbfit{Enterococcus}$'
    ax.set_title(title_string, fontsize=18)
    ax.set_xlabel('Antibiotics', fontsize=16)
    ax.set_ylabel('Strain ID (Species)', fontsize=16)
    ax.set_xticklabels(ax.get_xticklabels(), rotation=45, ha='right', fontsize=13)

    new_yticklabels = []
    for label_text in heatmap_data.index:
        parts = label_text.rsplit(' (', 1)
        if len(parts) == 2:
            strain_id = parts[0]
            species_name = parts[1][:-1]
            species_name_formatted = species_name.replace(' ', r'\ ')
            new_label_str = r'$\mathrm{' + strain_id + r'}\ (\mathit{' + species_name_formatted + r'})$'
            new_yticklabels.append(new_label_str)
        else:
            new_yticklabels.append(label_text)
    ax.set_yticklabels(new_yticklabels, fontsize=13)
```

```
    legend_labels = {
        '1: Phenotype and Genotype present': colors[0],
        '2: Phenotype and Genotype absent': colors[1],
        '3: Phenotype present, Genotype absent': colors[2],
        '4. Phenotype absent, Genotype present': colors[3],
        '5: Others': colors[4]
    }
    patches = [mpatches.Patch(color=color, label=label) for label, color in legend_labels.items()]

    legend = ax.legend(
        handles=patches,
        loc='center left',
        bbox_to_anchor=(1.02, 0.5),
        borderaxespad=0.,
        frameon=False,
        title='Category Explanation',
        fontsize=16,
        title_fontsize=18
    )
    plt.setp(legend.get_title(), weight='bold')

    output_filename_tif = "Enterococcus_Heatmap_Styled.tif"
    try:
        plt.savefig(output_filename_tif,     dpi=300,     format='tiff',     bbox_inches='tight',
pad_inches=0.5)
        print(f"\nHeatmap successfully saved as high-resolution TIF: {output_filename_tif}")
    except Exception as e:
        print(f"Error occurred while saving heatmap TIF file: {e}")

    plt.close()

if __name__ == '__main__':
    main()
```

Co-occurrence Network Analysis

```
    import pandas as pd
    import networkx as nx
    import re
    from collections import defaultdict
    from itertools import combinations

    def main():
        print("--- Starting data processing ---")
```

```
    excel_file_path = 'AMR gene co-occurrence.xlsx'
    try:
        df = pd.read_excel(excel_file_path)
        print(f"Successfully read the file: '{excel_file_path}'")
    except FileNotFoundError:
        print(f"Error: File '{excel_file_path}' not found.")
        return

    df.columns = [col.replace(' ', '_') for col in df.columns]
    required_cols = ['SampleID', 'AMR_Gene', 'antibiotic_class', 'Location_Detail']
    if not all(col in df.columns for col in required_cols):
        print("Error: Required columns are missing from the file.")
        return

    df.dropna(subset=['SampleID', 'AMR_Gene'], inplace=True)
    df['SampleID'] = df['SampleID'].astype(str).str.strip()
    df['AMR_Gene'] = df['AMR_Gene'].astype(str).str.strip()
    df['Location_Detail'] = df['Location_Detail'].astype(str).str.strip().str.capitalize()
    df['AMR_Class'] = df['antibiotic_class'].astype(str).str.strip().str.upper()

    print("Calculating gene co-occurrence frequencies...")
    sample_genes = df.groupby('SampleID')['AMR_Gene'].apply(lambda x:
sorted(list(set(x))))
    co_occurrence_counts = defaultdict(int)
    for genes in sample_genes:
        for gene1, gene2 in combinations(genes, 2):
            co_occurrence_counts[tuple(sorted((gene1, gene2)))] += 1

    print("Building the network and integrating attributes...")
    G = nx.Graph()
    threshold = 3

    print("Determining final gene locations (Plasmid/Chromosome/Both)...")
    gene_location_sets = df.groupby('AMR_Gene')['Location_Detail'].apply(set)
    gene_final_location = {}
    for gene, locations in gene_location_sets.items():
        clean_locations = {str(loc).capitalize() for loc in locations}
        if 'Plasmid' in clean_locations and 'Chromosome' in clean_locations:
            gene_final_location[gene] = 'Both'
        elif 'Plasmid' in clean_locations:
            gene_final_location[gene] = 'Plasmid Only'
        elif 'Chromosome' in clean_locations:
            gene_final_location[gene] = 'Chromosome Only'
        else:
```

```
                gene_final_location[gene] = 'Unknown'

        unique_genes_df = df[['AMR_Gene', 'AMR_Class']].drop_duplicates(subset=['AMR_Gene']).set_index('AMR_Gene')
        for gene_name, attrs in unique_genes_df.iterrows():
            G.add_node(
                gene_name,
                AMR_Class=attrs['AMR_Class'],
                Location_Detail=gene_final_location.get(gene_name, 'Unknown')
            )

        for (gene1, gene2), count in co_occurrence_counts.items():
            if count >= threshold:
                if G.has_node(gene1) and G.has_node(gene2):
                    G.add_edge(gene1, gene2, Weight=count)

        G.remove_nodes_from(list(nx.isolates(G)))

        weighted_degrees = dict(G.degree(weight='Weight'))
        nx.set_node_attributes(G, weighted_degrees, 'Weighted_Degree')

        components = list(nx.connected_components(G))
        cluster_map = {node: f'Cluster {i + 1}' for i, comp in enumerate(components) for node in comp}
        nx.set_node_attributes(G, cluster_map, 'Cluster_ID')

        if G.number_of_nodes() == 0:
            print("The network is empty. Aborting further processing.")
            return

        output_filename_graphml = "network_for_visualization.graphml"
        try:
            nx.write_graphml(G, output_filename_graphml)
            print(f"\nProcessing completed! Network file successfully generated: '{output_filename_graphml}'")
        except Exception as e:
            print(f"Error while exporting GraphML file: {e}")

    if __name__ == '__main__':
        main()
```

Plasmid-Resistance Gene Network

```
import pandas as pd
import networkx as nx
```

```
def main():
    print("--- Starting bipartite network data processing ---")

    try:
        df_amr = pd.read_excel('amr_data.xlsx')
        df_replicons = pd.read_excel('replicon_data.xlsx')
        print("Successfully read 'amr_data.xlsx' and 'replicon_data.xlsx'.")
    except FileNotFoundError as e:
        print(f"Error: Required Excel file not found. {e}")
        return

    # Standardize and clean input data
    df_amr['Contig'] = df_amr['Contig'].astype(str).str.strip()
    df_replicons['Contig'] = df_replicons['Contig'].astype(str).str.strip()
    df_amr['SampleID'] = df_amr['SampleID'].astype(str).str.strip()
    df_replicons['SampleID'] = df_replicons['SampleID'].astype(str).str.strip()

    # Merge the two datasets on SampleID and Contig
    df_merged = pd.merge(df_amr, df_replicons, on=['SampleID', 'Contig'], how='inner')
    if df_merged.empty:
        print("Warning: Merged dataset is empty. Cannot generate network. Please check the input files and merge logic.")
        return

    # Determine which column to use for AMR entity
    amr_entity_column = 'AMR_Gene_Class' if 'AMR_Gene_Class' in df_merged.columns else 'AMR_Gene'
    replicon_column = 'Detected Plasmid Replicon Type'
    print(f"Using '{amr_entity_column}' and '{replicon_column}' to construct the network.")

    # Create edge list based on co-occurrence counts
    edge_data = df_merged.groupby([amr_entity_column, replicon_column])['SampleID'].nunique().reset_index()
    edge_data = edge_data.rename(columns={'SampleID': 'Weight'})

    if edge_data.empty:
        print("No associations found. Cannot build network.")
        return

    print("Building network and assigning attributes...")
    B = nx.Graph()
```

```
        amr_nodes = pd.unique(edge_data[amr_entity_column])
        replicon_nodes = pd.unique(edge_data[replicon_column])

        for node in amr_nodes:
            B.add_node(node, type='AMR_Entity')
        for node in replicon_nodes:
            B.add_node(node, type='Replicon')

        for _, row in edge_data.iterrows():
            B.add_edge(row[amr_entity_column], row[replicon_column], Weight=row['Weight'])

        weighted_degrees = dict(B.degree(weight='Weight'))
        nx.set_node_attributes(B, weighted_degrees, 'Weighted_Degree')

        print(f"Network built: {B.number_of_nodes()} nodes, {B.number_of_edges()} edges.")

        output_filename_graphml = "bipartite_network.graphml"
        try:
            nx.write_graphml(B, output_filename_graphml)
            print("\nProcessing completed!")
            print(f"Final network file successfully generated: '{output_filename_graphml}'")
            print("You can now open this file in Cytoscape or Gephi for visualization.")
        except Exception as e:
            print(f"Error while exporting GraphML file: {e}")

    if __name__ == '__main__':
        main()
```

Heatmap of Plasmid and AMR Gene Types

```
import pandas as pd
import seaborn as sns
import matplotlib.pyplot as plt
import numpy as np

def main():
    print("--- Start processing heatmap data ---")

    try:
        df_amr = pd.read_excel('amr_data.xlsx')
        df_replicons = pd.read_excel('replicon_data.xlsx')
        print("Successfully loaded 'amr_data.xlsx' and 'replicon_data.xlsx'.")
    except FileNotFoundError as e:
```

```
            print(f"Error: Required Excel files not found. {e}")
            return

        required_amr_cols = ['SampleID', 'AMR_Gene', 'Contig']
        required_replicon_cols = ['SampleID', 'Detected Plasmid Replicon Type', 'Contig']
        if not all(col in df_amr.columns for col in required_amr_cols) or \
                not all(col in df_replicons.columns for col in required_replicon_cols):
            print("Error: Missing required columns in one or both files.")
            return

        for df in [df_amr, df_replicons]:
            for col in ['SampleID', 'Contig']:
                df[col] = df[col].astype(str).str.strip()

        df_merged = pd.merge(df_amr, df_replicons, on=['SampleID', 'Contig'], how='inner')
        if df_merged.empty:
            print("Warning: Merged data is empty. Cannot generate heatmap.")
            return

        print("Creating frequency matrix for heatmap...")
        row_variable = 'AMR_Gene_Class' if 'AMR_Gene_Class' in df_merged.columns else
'AMR_Gene'
        col_variable = 'Detected Plasmid Replicon Type'

        heatmap_data = pd.crosstab(
            df_merged[row_variable],
            df_merged[col_variable],
            values=df_merged['SampleID'],
            aggfunc='nunique'
        ).fillna(0).astype(int)

        if heatmap_data.empty:
            print("Crosstab result is empty. Cannot generate heatmap.")
            return

        print("Drawing heatmap...")

        plt.rcParams.update({
            'font.family': 'sans-serif',
            'font.sans-serif': ['Arial', 'Helvetica'],
            'axes.labelweight': 'bold',
            'axes.titleweight': 'bold',
            'axes.unicode_minus': False,
            'axes.labelsize': 16
```

```
    })

    fig_width = max(12, heatmap_data.shape[1] * 0.8)
    fig_height = max(10, heatmap_data.shape[0] * 0.6)
    plt.figure(figsize=(fig_width, fig_height))

    cbar_label_style = {'label': 'Number of Isolates with Association', 'shrink': 0.75}

    sns.heatmap(
        heatmap_data,
        annot=True,
        fmt="d",
        cmap="YlGnBu",
        linewidths=.5,
        linecolor='gray',
        cbar_kws=cbar_label_style,
        annot_kws={"size": 12}
    )

    plt.title(f'Association between {row_variable} and Plasmid Replicon Types', fontsize=20)
    plt.ylabel(row_variable)
    plt.xlabel(col_variable)
    plt.xticks(rotation=45, ha="right", fontsize=13)
    plt.yticks(rotation=0, fontsize=13)

    plt.tight_layout(pad=2.0)

    output_filename_tif = "AMR_Replicon_Association_Heatmap.tif"
    try:
        plt.savefig(output_filename_tif, dpi=300, format='tiff', bbox_inches='tight')
        print(f"\nHeatmap saved to: {output_filename_tif}")
    except Exception as e:
        print(f"Error saving heatmap: {e}")

    plt.show()

if __name__ == '__main__':
    main()
```
